# Increasing Physical Activity in Persons With Spinal Cord Injury With an eHealth-Based Adaptive Exercise Intervention: Protocol for a Sequential Multiple Assignment Randomized Trial

**DOI:** 10.2196/47665

**Published:** 2023-07-27

**Authors:** Jereme Wilroy, Yumi Kim, Byron Lai, Hui-Ju Young, John Giannone, Danielle Powell, Mohanraj Thirumalai, Tapan Mehta, James Rimmer

**Affiliations:** 1 Department of Physical Medicine and Rehabilitation University of Alabama at Birmingham Birmingham, AL United States; 2 Division of Pediatric Rehabilitation Medicine University of Alabama at Birmingham Birmingham, AL United States; 3 Research Collaborative University of Alabama at Birmingham Birmingham, AL United States; 4 Department of Health Services Administration University of Alabama at Birmingham Birmingham, AL United States; 5 Department of Family and Community Medicine University of Alabama at Birmingham Birmingham, AL United States

**Keywords:** exercise, physical activity, spinal cord injury, adaptive intervention

## Abstract

**Background:**

Participating in an adequate amount of physical activity to acquire health benefits is challenging for people with spinal cord injury (SCI) due to personal and logistic barriers. Barriers in the built and social environments may include lack of transportation, lack of accessible facilities or programs, and lack of training among fitness personnel. Low self-efficacy, lack of self-regulation skills, and improper outcome expectations are examples of personal barriers. Current approaches to investigating physical activity programs in people with SCI have been limited to traditional “one-size-fits-all” design, which has yielded low adherence rates, high dropout rates, and participants not maintaining physical activity levels at follow-up.

**Objective:**

The primary aim of this study is to test the feasibility of a tele-exercise program that applies an adaptive intervention design for 30 adults with SCI, targeting increases in adherence to the exercise program and physical activity participation.

**Methods:**

The Sequential Multiple Assignment Randomized Trial for Home-based Exercise and Lifestyle Tele-Health (SMART-HEALTH) is a 12-week, home-based, movement-to-music (M2M) program. The goal of a SMART-designed study is to develop an adaptive intervention that modifies support provisions based on response levels. In SMART-HEALTH, 2 groups of participants will undergo 3-week and 6-week asynchronous M2M interventions in the first phase. Participants who did not achieve the desired adherence rate (≥95% of video watch minutes) will be rerandomized into M2M Live (switch) or individualized behavioral coaching (augmented with the asynchronous M2M program). The study will primarily assess rates of recruitment or enrollment, adherence and retention, timing to identify nonresponders, and scientific outcomes (eg, physical activity and exercise self-efficacy). The study will qualitatively evaluate the acceptability of the study using semistructured interviews among participants who complete the 12-week intervention.

**Results:**

Recruitment procedures started in June 2022. All data are expected to be collected by September 2023. Full trial results are expected to be published by March 2024. Secondary analyses of data will be subsequently published. Results will include exercise adherence rates; changes in self-reported physical activity levels and blood pressure; and changes in secondary conditions including pain, sleep, and fatigue. Thematic analysis of semistructured interviews will include results on participant enjoyment and acceptability of SMART-HEALTH and inform modifications for future delivery of the program.

**Conclusions:**

This study will strengthen our understanding of the potential benefits of the tele-exercise intervention for people with SCI and build upon adaptive intervention design and its delivery strategies that aim to increase adoption and sustainable exercise behavior. This pilot trial will inform future SMART-designed studies and provide new and innovative strategies for investigating intervention effects on physical activity behavior in the SCI population.

**Trial Registration:**

ClinicalTrials.gov NCT04726891; https://classic.clinicaltrials.gov/ct2/show/NCT04726891

**International Registered Report Identifier (IRRID):**

DERR1-10.2196/47665

## Introduction

Approximately 302,000 individuals in the United States are living with spinal cord injury (SCI) [1]. Substantial evidence supports the benefits of exercise training for improving health and functional outcomes and managing the risk of chronic disease and secondary conditions related to the SCI (eg, cardiometabolic disease, obesity, and depression) [2-8]. Despite the benefits, people with SCI engage in considerably lower levels of exercise and physical activity participation compared with the general population of adults and individuals living with other health conditions [4,9,10]. This may have resulted from a multilevel of barriers that people with SCI experience with exercise and physical activity participation in the community (eg, a lack of accessible facilities or options, knowledgeable instructors, transportation, or social support) [9].

Furthermore, people with SCI, who initiated engaging in an exercise behavior, often have varying rates of success in standardized exercise interventions, which have been designed using a traditional “one-size-fits-all” approach (ie, exercise group vs control) [11,12]. Previous studies have often demonstrated low levels of adherence to exercise programs, high dropouts, and a large portion of the sample not continuing exercise behavior throughout the follow-up [13-15]. In addition, home-based exercise programs are a desirable approach for overcoming barriers and facilitating full participation that could be of great assistance to people with SCI. Tele-exercise programs are potentially more cost-efficient and convenient (ie, do not require transportation or large travel times) than programs offered in the community, making it easier to reach larger groups of people with SCI [16]. Collectively, these findings suggest that the design of future home-based exercise training studies should be more targeted and tailored interventions for people with SCI.

Adaptive interventions provide a flexible treatment regimen based on the participant’s evolving status and specific needs, previously used in healthy behavior promotion research (eg, smoking cessation and weight loss) [17-20]. Sequential Multiple Assignment Randomized Trial (SMART) is an experimental design that enables investigators to answer whether, when, and how to alter treatment intensity and type to build an evidence-based adaptive intervention [21]. The use of the SMART design has the potential to optimize the design and delivery strategies of an exercise promotion intervention for maximizing adherence to the program in people with SCI; yet we are unaware of exercise interventions applied and tested this design for people with SCI.

We further note the importance of embedding behavioral approaches to increase physical activity in people with SCI. Based on prior research [22], the study design and delivery are grounded in social cognitive theory (SCT) [23,24], which has been used successfully to improve many health behaviors, such as diet and exercise adherence, in individuals with SCI. The main concept of SCT is the interplay between personal, environmental, and behavioral factors [25]. Outcome expectations [26-29], self-efficacy [28,30-32], social support [26,28,30,31], and self-regulation [26,31,33] are key constructs of SCT that have been associated with changes in various health behaviors in past studies, including several studies involving a broader group of people with mobility disability [27,30,33,34].

We propose a pilot, 2-phase SMART examining the feasibility of a 12-week tele-exercise intervention for improving adherence to the program and physical activity behavior in 30 adults with SCI, named the Sequential Multiple Assignment Randomized Trial for Home-based Exercise And Lifestyle Tele-Health (SMART-HEALTH). The applied exercise intervention is a novel movement-to-music (M2M) video program that incorporates enjoyable movement routines accompanied by music to improve cardiovascular capacity, muscular strength and endurance, balance, and range of motion. Evidence supports the efficacy of M2M in enhancing functional mobility (the timed up and go and 6-minute walk test) among adults with multiple sclerosis [35]. Also, 1 ongoing tele-exercise trial is testing the effectiveness of the M2M video program that includes >400 adults with physical disabilities (NCT03024320) [36]. The modified and scaled-up M2M program was delivered at a state-of-the-art exercise facility for people with disabilities (Lakeshore Foundation). This program integrates the principles of SCT through weekly newsletters that contained educational information aligned with the key constructs of SCT (self-efficacy, outcome expectation, knowledge, self-regulatory strategies, facilitators, or barriers) for promoting and maintaining behavior change.

The primary aim of this study is to assess the feasibility of the 12-week tele-exercise intervention with the SMART design application. The assessment of the feasibility focuses on recruitment or enrollment, adherence, and retention rates, resources and management issues, timing to identify nonresponders, and adverse events (AEs). The secondary aim is to qualitatively assess the acceptability of the SMART-HEALTH intervention. Acceptability will be assessed qualitatively via thematic analysis of semistructured exit interviews conducted at 3 months from baseline, with participants who complete the pilot study. The tertiary aim is to estimate average intervention effects and variability on the scientific outcomes (eg, video watch minutes, self-reported vs objectively measured physical activity) to determine effect sizes and potential for a larger trial. The information yielded by such a feasibility study will be critical for designing larger-scale projects that can establish the actual efficacy and effectiveness of the future SMART-HEALTH study.

## Methods

### Study Design

**Figure 1:**
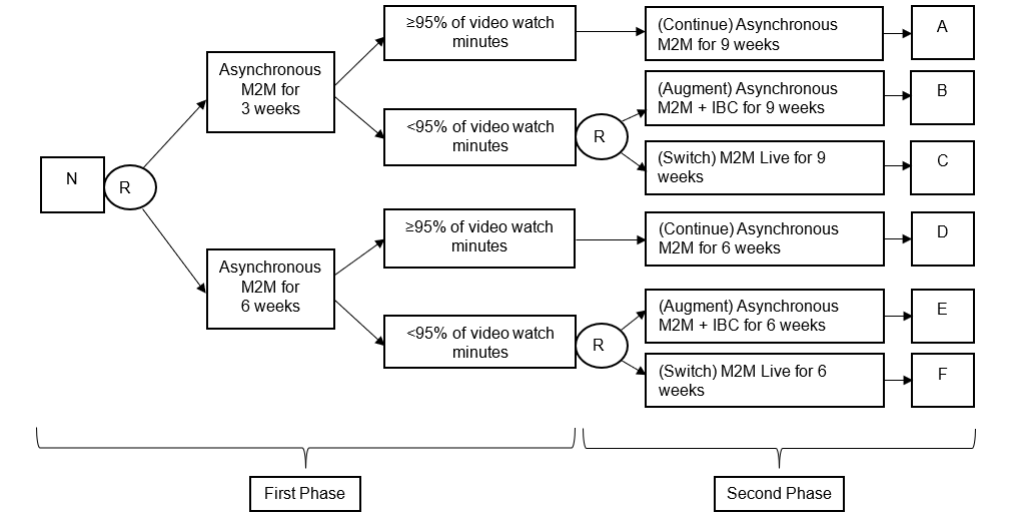
Sequential Multiple Assignment Randomized Trial for Home-based Exercise and Lifestyle Tele-Health (SMART-HEALTH) study design for people with spinal cord injury. IBC: individualized behavioral coaching; M2M: movement-to-music intervention; N: sample; R: randomize.

This pilot study applied a 2-phase SMART design. Thirty adults with SCI will be randomized with equal probability into 1 of 2 asynchronous, M2M exercise programs (using prerecorded videos) at the first phase of the intervention. The exercise minutes of the pre-recorded videos are set to progressively increase from 30 to 150 minutes per week throughout the 12 weeks. The response assessment will be administered at weeks 3 (group 1) and 6 (group 2) based on the predetermined adherence criteria to the intervention (average of 95% video watch minutes, ranging from 29 minutes to 143 minutes). Participants who meet or exceed the criteria will be considered as responders and continue the asynchronous program for the entire intervention period, whereas those who did not meet the criteria (nonresponders) will be rerandomized with equal probability into either synchronous, instructor-led M2M exercise program (ie, switch to M2M Live group) or behavioral coaching with asynchronous M2M exercise program (ie, augment with individual behavioral coaching; IBC). The study flow diagram includes the details of the interventions offered in each arm (Figure 1).

### Ethics Approval

Ethics approval was obtained from the institutional review board (IRB) of the University of Alabama at Birmingham on March 10, 2021 (IRB-300006746), and registered at ClinicalTrials.gov (NCT04726891). Informed consent will be obtained from all participants prior to the research activities. For privacy and confidentiality protection, all data will be collected and stored on a secure, web-based database application, referred to as Research Electronic Data Capture (REDCap; Vanderbilt University). Participants will receive up to US $160 of monetary incentives using direct deposit in a reloadable debit card (ie, ClinCard). This includes US $25 for baseline assessments, US $60 for weekly post workout surveys (US $5 for each survey×12 surveys), US $50 for post intervention assessments, and US $25 for an exit interview at the end of the 12-week intervention.

### Eligibility Criteria

The inclusion criteria are (1) age 18 years and older; (2) diagnosis of a traumatic SCI that occurred more than 12 months before enrollment; (3) ability to use the arms to exercise (ie, complete [C5 and below] or incomplete paraplegia and tetraplegia); (4) willingness and ability to participate in a 3-month home-based exercise program and study procedures (eg, having internet access, not having visual impairment that prevents watching exercise videos on a computer screen, having complete assessments available, ability to wear an accelerometer, and willingness to undergo multiple randomizations); (5) being physically inactive (defined as ≥150 minutes of moderate-to-vigorous intensity activities in a typical week) [37], and (6) having 1 or more affirmatives on the Physical Activity Readiness Questionnaire for Everyone (PAR-Q) [38] or physician approval for undertaking exercise training for those with 2 or more affirmatives on the PAR-Q. More than 1 affirmative on the PAR-Q indicates that the participant is at more than minimal risk for exercise-related complications, and therefore, physician approval will be required prior to enrolling such participants in this study. Exclusion criteria are (1) history (<6 months) of cardiovascular or pulmonary disease; (2) any unstable medical condition that is likely to compromise safety, like active pressure ulcer, pregnancy, or renal failure; and (3) any cognitive dysfunction or language barriers that would prevent participants from following English instructions.

### Recruitment and Enrollment

We reviewed our database and identified 1016 persons who fit our inclusion criteria (disability status and expressed interest to participate in future research). Additionally, we identified 347 people with spinal cord dysfunction through an external database. We sent out invitation emails to all potential participants across the United States.

We advertised the study information through social media channels and the Lakeshore Foundation. The Lakeshore Foundation has the ability to distribute an electronic newsletter for people with SCI and other disabilities, social media channels, community events, and through the National Center on Health, Physical Activity and Disability (a center funded by the US Centers for Disease Control and Prevention).

Interested individuals can contact the study staff via phone, email, or online application linked with REDCap. A digital form of informed consent is given to eligible participants to review the study details, signed, and submitted. A project coordinator, who is responsible for participant communications and process management, follow-ups with the prospective participant via phone call, verbally reviews the consent form, answers any study-related questions and concerns, and guides the following study procedures.

### Randomization Allocations and Other Trial Considerations

Participants are potentially randomized up to twice throughout the 12-week intervention period. A study statistician generated 3 randomization sequences a priori using a computer-generated random schedule (SAS version 9.4; SAS Institute) with a 1:1 allocation ratio and a permuted block approach. The sequences were stored into a randomization module in the REDCap and external program, which allows project staff to unfold the randomization schedule when needed (after completing baseline assessment and after a 3-week or 6-week check-in call). One sequence was used to perform the first randomization of 30 participants into one of the asynchronous M2M exercise groups (group 1 vs group 2), whereas other 2 sequences were used to perform the second randomization of participants who need a transition from asynchronous M2M exercise to M2M Live or IBC based on the adherence rate to the intervention. Response assessment (ie, adherence rate to the program) will be administered at week 3 and week 6, respectively, for the intervention groups 1 and 2. Participants identified as responders who watched the video more than 95% (accounting for 5% error rate) will continue with the first treatment regime. Participants will be considered nonresponders if they adhere to the exercise program less than 95% using video watch minutes. This is based on observation with a few weight loss studies that suggested sessions 3 and 7 as potential time points for intervening with participants to assess needs and difficulties and make necessary changes to maximize the treatment response [39-42].

The outcome assessor will be blinded to group allocation (single-blinded trial design). Due to the nature of the intervention, it is not plausible to blind participants and study staff who enroll participants and monitor the intervention delivery. However, study investigators and staff who are involved in data analyses and reporting will be blinded to group assignments throughout the study period.

Access to the REDCap database will be given to the study personnel only, including the biostatistician and data entry or management personnel. Data that are analyzed will be exported in a deidentified format. The identities of participants will not be revealed in the presentation or publication of any result from this project.

### Home-Based Intervention

#### Overview

The interventions will be delivered through Canvas, which is a secured, commercialized learning management system. First, all participants will receive weekly notifications when a new module is available on Canvas, including exercise videos and educational studies. The weekly notifications can be delivered through the Canvas system and email. Third, participants will be able to leave comments and questions on exercise videos, educational studies, and post workout comment section. Fourth, to ensure any questions or technical issues can be addressed in a timely manner, participants will be able to reach the study team via multiple communication channels, including a study phone number, email, or messaging via the Canvas system.

#### Prerecorded, Asynchronous M2M Videos

Both groups of participants receive the same exercise prescriptions at the first phase of the intervention. The M2M program provides a sequence of exercise routine, which is accompanied by music to improve range of motion, muscular strength and endurance, cardiovascular capacity, and balance or proprioception. The weekly exercise video is set to progressively increase the exercise minutes to enable the participants to reach and exceed the recently published SCI exercise guideline [38]. Both groups of participants will begin with 30 minutes of exercise (twice weekly or 15 minutes per session) and increase the minutes incrementally until they reach the SCI guideline of 90 minutes at week 6 (3 times weekly or 30 minutes per session) and 150 minutes at week 9 (3 times weekly or 50 minutes per session). The detailed exercise prescription is presented in Table 1.

**Table 1:** Description of exercise components and prescriptions in minutes per week.

Exercise Component	Week 1, minutes	Week 2, minutes	Week 3, minutes	Week 4, minutes	Week 5, minutes	Week 6, minutes	Week 7, minutes	Week 8, minutes	Week 9, minutes	Week 10, minutes	Week 11, minutes	Week 12, minutes
ROM I^a^	5	5	5	5	5	N/A^b^	N/A	N/A	N/A	N/A	N/A	N/A
ROM II	N/A	N/A	N/A	5	5	5	5	5	5	5	5	5
Muscular Strength I	5	5	5	5	N/A	N/A	N/A	N/A	N/A	N/A	N/A	N/A
Muscular Strength II	N/A	N/A	N/A	N/A	5	5	5	5	N/A	N/A	N/A	N/A
Muscular Strength III	N/A	N/A	N/A	N/A	N/A	N/A	N/A	N/A	5	5	5	5
Aerobic I	N/A	N/A	N/A	5	5	N/A	N/A	N/A	N/A	N/A	N/A	N/A
Aerobic II	N/A	N/A	N/A	N/A	N/A	10	10	10	10	10	10	10
Aerobic III	N/A	N/A	N/A	N/A	N/A	N/A	N/A	N/A	10	10	10	10
Aerobic IV	N/A	N/A	N/A	N/A	N/A	N/A	N/A	10	10	10	10	10
FSB I^c,d^	5	5	5	5	N/A	N/A	N/A	N/A	N/A	N/A	N/A	N/A
FSB II^c,d^	N/A	N/A	N/A	N/A	5	5	5	5	N/A	N/A	N/A	N/A
FSB III^c,d^	N/A	N/A	N/A	N/A	N/A	N/A	N/A	N/A	5	5	5	5
Cooldown	N/A	5	5	5	5	5	5	5	5	5	5	5
Total exercise per session	15	20	20	30	30	30	30	40	50	50	50	50
Total exercise per week	30	40	40	60	60	90	90	120	150	150	150	150

^a^ROM: range of motion.

^b^N/A: not available.

^c^FSB: functional strength and balance.

^d^Participants will be instructed to repeat the prerecorded videos 2 times per week for the first 5 weeks (week 1-5) and then increase the repetition to 3 times per week for the second 7 weeks (weeks 6-12).

At week 3 and week 6, adherence to the exercise program will be administered using Canvas video analytics. Participants who did not adhere to more than 95% of prescribed video watch minutes will be considered as nonresponders. These individuals will be communicated with by research staff at a prescheduled check-in date and guided to the changes of the program at the second phase intervention period:

#### IBC Group

Participants who are allocated into the IBC group will receive the remaining asynchronous M2M exercise prescriptions and augmented with weekly behavioral coaching. The first session was 50 minutes, and the remaining sessions were approximately 15 minutes. The coaching is designed to improve self-regulatory skills based on prior research and help them stick to the exercise prescription. An example of tailoring the exercise prescribed involves setting a goal of completing the exercise routine 1 time for the upcoming week instead of 3 times. The coach will be trained in motivational interviewing to help the participants modify their exercise habits. Telehealth coaching alone has been shown to increase self-reported physical activity in adults with SCI.

#### Synchronous, Instructor-Led M2M Program

Participants who are allocated into M2M Live will switch to one-on-one, synchronous tele-exercise training with an M2M instructor. M2M Live provides accountability and immediate, tailored feedback along with custom movements and music. Videoconference-delivered exercise has been shown as feasible among adults with SCI. During the first session of M2M Live, the participant will meet 1 time per week with the M2M instructor and perform an exercise session. The session will be video recorded, so participants can go back and complete the remaining sessions of the week.

#### Educational Newsletter

All participants will receive an educational newsletter per week, discussing health behavior change strategies such as goal-setting, planning, and coping with barriers. The purpose is to educate participants on self-regulation strategies and health-promoting behaviors.

### Remote Study Procedures

All study procedures, including screening, consent, data collection, and intervention, are conducted remotely at home. Survey data are collected through REDCap. Participants who complete baseline assessments will receive all equipment via mail and will be randomly allocated to 1 of the 2 M2M interventions. After randomization, each participant will receive an invitation email with a link to the Canvas platform, instructed to create a password, and schedule a check-in date at week 3 or 6 based on the group assignment. Participants will be instructed to perform the exercise program at least 3 times per week. Data collection equipment includes: (1) Chromebook (Lenovo Chromebook or Acer Chromebook 15); (2) Fitbit (Inspire 2, Fitbit); (3) Digital peak flow meter (Peak Flow Meter SMPF-1 and SONMOL); (4) blood pressure monitor (Omron 3 Series Upper Arm, Omron); (5) Two 454-gram wrist weights (Neoprene, thumb hole weights, Biicoon).

### Outcomes

#### Aim 1: Feasibility Metrics

All feasibility outcomes will be collected throughout the 12-week study period. This study will gather metrics of feasibility, including recruitment or enrollment (eg, frequency of contact with potential participants and recruitment sources, with questions such as, Where did you hear about our study? and the percentage of individuals who follow through the enrollment procedure); adherence and retention rates (eg, video watch minutes, attendance of M2M Live or IBC coaching sessions); resources and management issues (eg, preparation and completion time in minutes for functional outcome assessment; time in minutes for survey assessment); timing to identify nonresponders; and AE. The study team will monitor AEs throughout the study period and report them followed by the Behavior Change Consortium of the National Institutes of Health. The four types of AEs are (1) falls, (2) cardiovascular-related episodes, (3) musculoskeletal-related events, and (4) health care use. The severity and causality of each AE will be assessed and will be reported to IRB and relevant regulatory parties when necessary.

#### Aim 2: Acceptability

Acceptability will be assessed qualitatively via thematic analysis of semistructured exit interviews conducted at 3 months from baseline with participants who complete the pilot study.

#### Aim 3: Scientific Outcomes

Table 2 shows the physical and psychosocial outcomes, the schedule of data collection, and instruments used.

**Table 2:** Measures, instruments, and time points.

Variables and instruments			Time point
**Exercise adherence**			
	Tracking exercise video watch minutes (percentage)	12-week intervention period	
**Physical activity**			
	Leisure Time Physical Activity Questionnaire for People with Spinal Cord Injury	Pre or post	
	Postworkout survey	12-week intervention period	
**Lung function**			
	Spirometry	Pre or post	
**Pain intensity**			
	NIH^a^ PROMIS^b^ Pain Intensity Adult Short Form 3a	Pre or post	
**Pain interference**			
	NIH PROMIS Pain Interference Adult Short Form 8a	Pre or post	
**Sleep quality**			
	NIH PROMIS Sleep Disturbance Adult Short Form 8a	Pre or post	
**Fatigue**			
	NIH PROMIS Fatigue Adult Short Form 7a	Pre or post	
**Health-related quality of life**			
	NIH PROMIS 10 Global Health Items	Pre or post	
	NIH PROMIS Ability to Participate in Social Roles and Activities Short Form 8a	Pre or post	
**Exercise enjoyment**			
	Physical Activity Enjoyment Scale	Post	
**Social cognitive theory constructs**			
	Exercise Self-Efficacy Scale	Pre or post	
	Multidimensional Outcomes Expectations for Exercise Scale	Pre or post	
	Exercise Goal-setting and Planning Scale	Pre or post	
	Barriers in Physical Activity Questionnaire–Mobility Impairment	Pre or post	
	Social Provisions Scale	Pre or post	
**Demographics and health history**			
	Questionnaire	Pre	
**Blood pressure**			
	Blood pressure monitor	Pre or post	
**App quality and usability**			
	Systems Usability Scale	Post	

^a^NIH: National Institutes of Health.

^b^Patient-Reported Outcomes Measurement Information Systems.

##### Adherence to the Exercise Program

Video statistics (as a percentage) will be recorded through a secured, commercialized learning management system.

##### Physical Activity

Physical activity will be administered using both objective and self-reported measures. Objective physical activity will be measured using Fitbit throughout the 12-week intervention period, while self-reported physical activity will be measured using the weekly Leisure Time Physical Activity Questionnaire for People With SCI.

##### Postworkout Survey

Weekly survey will be administered within the learning management system to capture the perceived intensity of exercise and any verbatim comments to the program.

##### Physiological Function

All functional teleassessment will be conducted via videoconferencing calls (teleassessments). Data collection toolkits (eg, blood pressure monitors and spirometry) will be mailed to the participants’ location upon the completion of randomization. The data collection will take approximately half an hour and led by an exercise physiology specialist.

##### Psychosocial Health

Psychosocial health outcomes will include pain, sleep, fatigue, perceived exercise enjoyment, quality of life, and SCT constructs. These outcomes will be collected via REDCap. All self-report data will be collected directly through electronic questionnaire packets delivered via REDCap at baseline and post intervention assessments. All question items of the questionnaire packets are made as required to answer to prevent any missing data. Each questionnaire packet is also set to be delivered to participants’ email 3 times, within a span of 3 days if it is not completed. Research staff will be notified via REDCap when the third packet was sent out to participants. A follow-up phone call will be made to ensure participants receive the packet and remind them to complete it within the next 3 days. Packets that are incomplete within the 3 days after the phone call will be closed and recorded as incomplete, missing data.

### Analysis

#### Overview

All data will be exported and analyzed using SPSS (version 22.0; IBM). Data will be initially examined for variations, outliers, errors, and patterns of missing values. Missing data will be inputted using multiple imputation techniques where necessary based on the assumption that the missingness mechanism is at random. All statistical analyses will be conducted in an intent-to-treat manner at the individual level.

#### Aim 1

The primary outcomes of this pilot study will include enrollment rate, retention rate, and completion rate of surveys and physiological measures. Process, resource, and management feasibility will initially be examined via percentage, frequency analysis, and descriptive statistics.

#### Aim 2

A qualitative study of participants’ experiences, perceptions, and suggestions regarding the SMART-HEALTH program will be completed at week 12. This study will refine the study components and implementation processes for a future trial. In brief, participants who complete the SMART-HEALTH program will take part in single, one-on-one semistructured interviews with questions focusing on what worked and did not work. Interviews will be audio-recorded, transcribed, and analyzed using thematic analysis using a qualitative descriptive design [43].

#### Aim 3

The study will estimate recruitment and retention rates, as well as estimate outcomes and variability of efficacy outcomes for a larger clinical trial (aim 3), which will measure rates of physical activity, psychosocial variables (eg, depression), and physiological variables (eg, lung function). Additional fidelity measures will include adherence to exercise intervention (minutes of sessions viewed), number of coaching sessions completed, and intervention safety (number of AEs).

### Power

We will aim to enroll at least 40 people with SCI, considering 25% of the attrition rate based on the preliminary data obtained in the exercise intervention study with the M2M program [44]. The sample size of 30 participants aligns with the statistical recommendations for pilot trials to determine precise estimates, and the expectation that at least 5 participants will move each rerandomization group aligns with the statistical recommendations for the pilot SMART trial to determine precise estimates [45,46].

## Results

This study was approved by the university IRB on March 10, 2021, and initiated on May 1, 2021. Recruitment started on June 22, 2022, and the first participant was enrolled on June 15, 2022. The trial is expected to be completed in September 2023.

## Discussion

### Principal Findings

This paper has presented the background and design for a SMART-HEALTH study investigating the feasibility of a home-based M2M exercise program, using an adaptive design for adults with SCI. Exercise is a critical health-enhancing behavior; yet, substantial evidence supports various challenges of engaging in the recommended physical activity levels for many people with SCI. Tele-exercise programs may be suitable to overcome logistic barriers to exercise participation (eg, lack of transportation, travel time, and accessible facilities) and potentially more cost-efficient and convenient than programs offered in the community [16]. In addition, the application of the adaptive design to exercise intervention allows investigators to provide more tailored and targeted program delivery for people with SCI based on their needs while maintaining systematic approaches and scientific rigor. The ultimate goal of the SMART-HEALTH study is to find effective strategies to help guide and shape the exercise behavior of people with SCI toward higher levels of adoption and sustainable exercise participation. The SMART-HEALTH study will test multiple sets of adaptive intervention designs and their delivery strategies to optimize future intervention.

### Strength and Limitations

The trial uses an innovative study design to address adherence to exercise interventions among people with SCI. It addresses a growing need among physicians treating patients with mobility disabilities to have easily accessible eHealth home-based exercise videos that can be tailored to the functional level of their patients. This is a pilot trial to assess the feasibility of using a SMART design and is not powered for effectiveness.

### Conclusions

The trial serves to inform the development of adaptive interventions, including whether, how, or when to alter treatment intensity, type, or delivery. In addition, this can determine the actual efficacy of home-based exercise based on the SCI-specific physical activity programs for improving adherence to the program and physical activity.

## Appendix

Multimedia Appendix 1 Peer-review report by the Neilsen Foundation (USA).

## Abbreviations

AE: adverse event
IBC: individual behavioral coaching
IRB: institutional review board
M2M: movement-to-music
PAR-Q: Physical Activity Readiness Questionnaire for Everyone
REDCap: Research Electronic Data Capture
SCI: spinal cord injury
SCT: social cognitive theory
SMART: Sequential Multiple Assignment Randomized Trial
SMART-HEALTH: Sequential Multiple Assignment Randomized Trial for Home-based Exercise and Lifestyle Tele-Health

## Data Availability

The data generated as part of this study will be made available through a data repository. Deidentified data sets will be shared upon reasonable request.

## References

[1] (2020). Traumatic spinal cord injury facts and figures at a glance. National Spinal Cord Injury Statistical Center.

[2] Galea MP (2012). Spinal cord injury and physical activity: preservation of the body. Spinal Cord.

[3] Rimmer JH, Schiller W, Chen MD (2012). Effects of disability-associated low energy expenditure deconditioning syndrome. Exerc Sport Sci Rev.

[4] Carroll DD, Courtney-Long EA, Stevens AC, Sloan ML, Lullo C, Visser SN, Fox MH, Armour BS, Campbell VA, Brown DR, Dorn JM, Centers for Disease ControlPrevention (CDC) (2014). Vital signs: disability and physical activity--United States, 2009-2012. MMWR Morb Mortal Wkly Rep.

[5] Tomasone JR, Wesch NN, Ginis KAM, Noreau L (2013). Spinal cord injury, physical activity, and quality of life: a systematic review. Kinesiol Rev.

[6] Froehlich-Grobe K, Jones D, Businelle MS, Kendzor DE, Balasubramanian BA (2016). Impact of disability and chronic conditions on health. Disabil Health J.

[7] Lai B, Young HJ, Bickel CS, Motl RW, Rimmer JH (2017). Current trends in exercise intervention research, technology, and behavioral change strategies for people with disabilities: a scoping review. Am J Phys Med Rehabil.

[8] Rimmer JH, Chen MD, Hsieh K (2011). A conceptual model for identifying, preventing, and managing secondary conditions in people with disabilities. Phys Ther.

[9] Martin Ginis KA, Ma JK, Latimer-Cheung AE, Rimmer JH (2016). A systematic review of review articles addressing factors related to physical activity participation among children and adults with physical disabilities. Health Psychol Rev.

[10] Physical activity participation levels. Spinal Cord Injury Research Evidence.

[11] Ma JK, Martin Ginis KA (2018). A meta-analysis of physical activity interventions in people with physical disabilities: content, characteristics, and effects on behaviour. Psychol Sport Exerc.

[12] Tomasone JR, Flood SM, Ma JK, Scime NV, Burke SM, Sleeth L, Marrocco S, The SCIRE Research Team (2018). Physical activity self-management interventions for adults with spinal cord injury: part 1–a systematic review of the use and effectiveness of behavior change techniques. Psychol Sport Exerc.

[13] Froehlich-Grobe K, Lee J, Aaronson L, Nary DE, Washburn RA, Little TD (2014). Exercise for everyone: a randomized controlled trial of project workout on wheels in promoting exercise among wheelchair users. Arch Phys Med Rehabil.

[14] Lai BC, Cederberg K, Vanderbom KA, Bickel CS, Rimmer JH, Motl RW (2018). Characteristics of adults with neurologic disability recruited for exercise trials: a secondary analysis. Adapt Phys Activ Q.

[15] Lai B, Kim Y, Wilroy J, Bickel CS, Rimmer JH, Motl RW (2019). Sustainability of exercise intervention outcomes among people with disabilities: a secondary review. Disabil Rehabil.

[16] Rintala A, Hakala S, Paltamaa J, Heinonen A, Karvanen J, Sjögren T (2018). Effectiveness of technology-based distance physical rehabilitation interventions on physical activity and walking in multiple sclerosis: a systematic review and meta-analysis of randomized controlled trials. Disabil Rehabil.

[17] Collins LM, Nahum-Shani I, Almirall D (2014). Optimization of behavioral dynamic treatment regimens based on the sequential, multiple assignment, randomized trial (SMART). Clin Trials.

[18] Fu SS, Rothman AJ, Vock DM, Lindgren B, Almirall D, Begnaud A, Melzer A, Schertz K, Glaeser S, Hammett P, Joseph AM (2017). Program for lung cancer screening and tobacco cessation: study protocol of a sequential, multiple assignment, randomized trial. Contemp Clin Trials.

[19] Noser AE, Cushing CC, McGrady ME, Amaro CM, Huffhines LP (2017). Adaptive intervention designs in pediatric psychology: the promise of sequential multiple assignment randomized trials of pediatric interventions. Clin Pract Pediatr Psychol.

[20] Sherwood NE, Butryn ML, Forman EM, Almirall D, Seburg EM, Lauren Crain A, Kunin-Batson AS, Hayes MG, Levy RL, Jeffery RW (2016). The BestFIT trial: a SMART approach to developing individualized weight loss treatments. Contemp Clin Trials.

[21] Almirall D, Nahum-Shani I, Sherwood NE, Murphy SA (2014). Introduction to SMART designs for the development of adaptive interventions: with application to weight loss research. Transl Behav Med.

[22] Wilroy J, Knowlden A (2016). Systematic review of theory-based interventions aimed at increasing physical activity in individuals with spinal cord injury. Am J Health Educ.

[23] Bandura A, Marks D (1986). Social foundations of thought and action. The Health Psychology Reader.

[24] Bandura A, Freeman WH, Lightsey R (1999). Self-efficacy: the exercise of control. J Cogn Psychother.

[25] Glanz K, Rimer BK, Viswanath K (2008). Health Behavior and Health Education: Theory, Research, and Practice, 4th edition.

[26] Kinnett-Hopkins D, Motl RW (2016). Social cognitive correlates of physical activity in black individuals with multiple sclerosis. Arch Phys Med Rehabil.

[27] Morrison JD, Stuifbergen AK (2014). Outcome expectations and physical activity in persons with longstanding multiple sclerosis. J Neurosci Nurs.

[28] Phillips SM, McAuley E (2013). Social cognitive influences on physical activity participation in long-term breast cancer survivors. Psychooncology.

[29] Suh Y, Joshi I, Olsen C, Motl RW (2014). Social cognitive predictors of physical activity in relapsing-remitting multiple sclerosis. Int J Behav Med.

[30] Anderson ES, Wojcik JR, Winett RA, Williams DM (2006). Social-cognitive determinants of physical activity: the influence of social support, self-efficacy, outcome expectations, and self-regulation among participants in a church-based health promotion study. Health Psychol.

[31] Anderson-Bill ES, Winett RA, Wojcik JR (2011). Social cognitive determinants of nutrition and physical activity among web-health users enrolling in an online intervention: the influence of social support, self-efficacy, outcome expectations, and self-regulation. J Med Internet Res.

[32] White SM, Wójcicki TR, McAuley E (2012). Social cognitive influences on physical activity behavior in middle-aged and older adults. J Gerontol B Psychol Sci Soc Sci.

[33] Motl RW, Dlugonski D, Wójcicki TR, McAuley E, Mohr DC (2011). Internet intervention for increasing physical activity in persons with multiple sclerosis. Mult Scler.

[34] Suh Y, Motl RW, Olsen C, Joshi I (2015). Pilot trial of a social cognitive theory-based physical activity intervention delivered by nonsupervised technology in persons with multiple sclerosis. J Phys Act Health.

[35] Young HJ, Mehta TS, Herman C, Wang F, Rimmer JH (2019). The effects of M2M and adapted yoga on physical and psychosocial outcomes in people with multiple sclerosis. Arch Phys Med Rehabil.

[36] Rimmer JH, Mehta T, Wilroy J, Lai B, Young HJ, Kim Y, Pekmezi D, Thirumalai M (2019). Rationale and design of a scale-up project evaluating responsiveness to home exercise and lifestyle tele-health (SUPER-HEALTH) in people with physical/mobility disabilities: a type 1 hybrid design effectiveness trial. BMJ Open.

[37] Piercy KL, Troiano RP, Ballard RM, Carlson SA, Fulton JE, Galuska DA, George SM, Olson RD (2018). The physical activity guidelines for Americans. J Am Med Assoc.

[38] Warburton DER, Bredin SSD, Jamnik VK, Gledhill N (2011). Validation of the PAR-Q+ and ePARmed-X+. Health Fitness J Can.

[39] Jeffery RW, Wing RR, Sherwood NE, Tate DF (2003). Physical activity and weight loss: does prescribing higher physical activity goals improve outcome?. Am J Clin Nutr.

[40] Jeffery RW, Wing RR, Thorson C, Burton LR (1998). Use of personal trainers and financial incentives to increase exercise in a behavioral weight-loss program. J Consult Clin Psychol.

[41] Wing RR, Jeffery RW (2001). Food provision as a strategy to promote weight loss. Obes Res.

[42] Wing RR, Jeffery RW (2003). Prescribed "breaks" as a means to disrupt weight control efforts. Obes Res.

[43] Clarke V, Braun V, Hayfield N (2015). Thematic analysis. Qualitative Psychology: A Practical Guide to Research Methods.

[44] Wilroy JD, Lai B, Davlyatov G, Mehta T, Thirumalai M, Rimmer JH (2021). Correlates of adherence in a home-based, self-managed exercise program tailored to wheelchair users with spinal cord injury. Spinal Cord.

[45] Whitehead AL, Julious SA, Cooper CL, Campbell MJ (2016). Estimating the sample size for a pilot randomised trial to minimise the overall trial sample size for the external pilot and main trial for a continuous outcome variable. Stat Methods Med Res.

[46] Almirall D, Compton SN, Gunlicks-Stoessel M, Duan N, Murphy SA (2012). Designing a pilot sequential multiple assignment randomized trial for developing an adaptive treatment strategy. Stat Med.

