# Supplementary material for: Increasing Physical Activity in Persons With Spinal Cord Injury With an eHealth-Based Adaptive Exercise Intervention: Protocol for a Sequential Multiple Assignment Randomized Trial
Source: JMIR Res Protoc. 2023 Jul 27;12:e47665. doi: 10.2196/47665 (PMC10415946; doi:10.2196/47665)
Supplement: Multimedia Appendix 1 [file resprot_v12i1e47665_app1.pdf]

Applicant: [redacted]
Title: Feasibility of the First Known Adaptive Intervention Delivering Innovative Exercise Program Optimized for People with SCI
Program: PSR Pilot Grants (PSR2-17)
Institution: The University of Alabama at Birmingham
App #: 645335

View Review Information

Close Window Print

Committee: PSR 2021 FGA

Summary Statement

Text Admin Summary:

The Neilsen Foundation has conditionally approved funding of this proposal. Enthusiasm for this Pilot project centered on its adaptive design. Reviewers commended the use of a stepped care model and the real-time use of social networking and Web-based behavioral coaching. Please see the reviewer critiques for more discussion of strengths and weaknesses.

Reviewer Role: Primary (19883)

OVERALL IMPACT

After considering all of the review criteria, summarize the significant strengths and weaknesses of the application. Will this project help establish a junior PI's research program and/or enhance an established program? Does the project have the potential for "high gain" to balance risk? Does the project scope suit the funding level and does it have the potential to exert a sustained powerful influence and/or be an important contribution to the field of SCI psychosocial research?

:

Strengths

- The SMART – adaptive trial design for tailoring the exercise program is innovative and well suited to overcoming traditional challenges associated with most physical activity trials.
- The trial incorporates virtual assessments and intervention which both accommodates the realities of the pandemic, but

also addresses challenges with in-person trials with respect to retention and overcoming barriers to participation and eventual reach of the intervention.
- This is a well-described, logical research design.
- This work brings together evidence and prior work by investigators (including recent work supported by Neilsen Foundation initiated when applicant was a postdoctoral applicant) into a comprehensive, novel intervention
- There is clarity in describing how the underlying theoretical basis (social cognitive theory) is operationalized in the Move 2 Music exercise intervention i.e., these constructs are embedded through the coaching and social networking approach.
- Additionally, there is attention paid to incorporating several behavioural features within the intervention (i.e., motivational interviewing).
- Good identification of potential problems and mitigation
- Exceptional facilities and strong team

Weaknesses
- Data analysis plan for secondary measures lacking (e.g., what is going to be done with Actigraph data?). It would be helpful if there were more information presented on what the specific methods are going to be for the qualitative analysis beyond “thematic analysis”. A little bit more information on the analytic approaches for the secondary measures of physical activity rates, sleep, depression, anxiety, pain, etc would have also been helpful beyond simply saying the variability and outcome effects will be estimated for a larger future trial.
- The description on facilities and resources seems impressive, but a little excessive, especially given little emphasis is placed on what the data analysis is actually going to consist of beyond looking at the primary outcomes associated with feasibility, acceptance, etc.

SIGNIFICANCE
1. Does the project address an important problem or a critical barrier in the field?

2. If the Aims of the project are achieved, how would this work change or enhance current methods, technologies, treatments, services, or interventions?

:
Physical activity is a very significant issue for persons with SCI and the proposal initiative represents a well-designed pilot bringing forward an innovative use of adaptive trial design which will enable a somewhat personalized approach for this issue. Adaptive trial designs seem to be a particularly useful approach to overcome some of the significant challenges for mounting physical activity trials in persons with SCI. Most notably, the potential for personalizing / tailoring interventions with this approach and still include the capability for a controlled experimental approach is a significant advance for the field. The proposed approach is an early attempt at incorporating these methods and should provide significant learnings for the further development of this approach.

RELEVANCE
1. How is this project relevant to the mission of the Neilsen Foundation?

2. How is this project relevant to the goal of the PSR portfolio?

:

This is an application that is very relevant to and consistent with both the Neilsen Foundation and the PSR portfolio – given the stated intent to enhance physical activity participation through a tailored intervention that also incorporates remote monitoring and programming. Several behavioural components are embedded in the both the interventional approach as well as the evaluative components which aligns well with the PSR portfolio.

INVESTIGATOR(S)

1. Are the PI, collaborators, and other contributors well suited to the project? Are relevant SCI and psychosocial research expertise represented on the proposed project team?

2. If the PI is a junior investigator, does he/she have appropriate experience, training and facilities to do the proposed work? If the PI is an established investigator, has he/she demonstrated an ongoing record of accomplishments that have advanced their field(s)?

:

This is a strong team bringing together a junior investigator showing promise along with an established mentor working within a setting that is state of the art and a leader in the field. There is a good track record between the 2 investigators featured on the application and also a solid support staff (i.e., biostatistician, technical support, exercise leaders, qualitative researcher, albeit the latter is junior and the description of qualitative methods was somewhat lacking in the proposal).

INNOVATION

1. Does the project challenge and seek to shift current research or clinical practice/interventional paradigms by utilizing novel theoretical concepts, approaches or methodologies, instrumentation, or interventions?

2. And/or does this application apply concepts, approaches or methodologies, instrumentation, or interventions from another field of research to SCI?

3. And/or is a refinement, improvement, or new application of theoretical concepts, approaches or methodologies, instrumentation, or interventions proposed?

:

The key innovation lies in bringing together various behavioural approaches within an adaptive trial methodology where both the intervention and the evaluation is enabled through remote technologies.
In particular, the adaptive trial methodology, as a means to support generation of research evidence for physical activity is an innovation that is important for the field in terms of developing more personalized approaches to enhancing physical activity participation.

APPROACH

1. Is the project well-suited to the Pilot stage? Are the future applicability or next steps after the Pilot work discussed (i.e., does this research help lay the groundwork for future studies or describe plans for the dissemination and translation of knowledge that will impact current practices and approaches)?

2. Are the overall strategy, methodology, and analyses well-reasoned and appropriate to accomplish the specific Aims of the project, within the proposed project period and by the project team described? Are potential problems, alternative strategies, and benchmarks for success presented?

3. If the project is in the early stages of development, will the strategy establish feasibility and will particularly risky aspects be managed adequately?

4. If the project involves clinical and/or community-based research:
a. Does the project timeline include plans for study start-up time and sufficient subject recruitment?
b. Does the start-up plan adequately address regulatory approval, if required?
c. Will the National Institute of Neurological Disorders and Stroke, Common Data Elements be used? If not, is justification provided?

:
The initiative is well-suited to the pilot stage. It is based on prior work (including recent work supported by Neilsen Foundation) into a comprehensive intervention that will be further developed and tested as a pilot. The methodology appears sound and well-suited to the pilot and there are several plans and mitigation strategies suggested to address feasibility for a successful trial.
The SMART – adaptive trial design for tailoring the exercise program is innovative and well suited to overcoming traditional challenges associated with most physical activity trials.
In addition, the trial incorporates virtual assessments and intervention which both accommodates the realities of the pandemic, but also addresses challenges with in-person trials with respect to retention and overcoming barriers to participation and eventual reach of the intervention.
In general, this is a well-described, logical research design.
There is clarity in describing how the underlying theoretical basis (social cognitive theory) is operationalized in the Move 2 Music exercise intervention i.e., these constructs are embedded through the coaching and social networking approach. Additionally, there is attention paid to incorporating several behavioural features within the intervention (i.e., motivational interviewing).

The data analysis plan for secondary measures lacking (e.g., what is going to be done with Actigraph data?). It would be helpful if there were more information presented on what the specific methods are going to be for the qualitative analysis beyond "thematic analysis". A little bit more information on the analytic approaches for the secondary measures of physical activity rates, sleep, depression, anxiety, pain, etc would have also been helpful beyond simply saying the variability and outcome effects will be estimated for a larger future trial.

ENVIRONMENT
1. Will the institutional environment in which the work will be done contribute to the probability of success?

2. Are the institutional support, physical equipment and other resources available to the investigator(s) adequate for the project proposed?

3. Will the project benefit from unique features of their environment, subject populations, or collaborative

arrangements?

:
The investigators have all the resources and support needed to conduct this initiative successfully. There is ample evidence within this environment of successfully implementing large research trials – and in particular all resources are in place along with a strong track record of supporting both physical activity trials including those that incorporate remote technologies. If anything, the description on facilities and resources, albeit impressive, seems a little excessive, especially given little emphasis is placed on what the secondary data analyses are actually going to consist of beyond looking at the primary outcomes associated with feasibility, acceptance, etc. (i.e., for both qualitative and quantitative secondary measures).

NON-SCORED CRITERIA
(PLEASE NOTE THAT THE SCORE FOR THIS NON-SCORED SECTION MUST BE A "50 NON-SCORED." PLEASE DISREGARD SCORING OPTIONS 1-9 FOR THIS SECTION ONLY.)

Provide any important or relevant comments on each of the non-scored criterion below.

1. Budget

2. Ethics/Safety; protection of human subjects, including safety and data monitoring

3. Other/Additional Comments for the Applicant

4. Resubmission: When reviewing a resubmission, the committee will evaluate the application as now presented, taking into consideration the responses to comments from the previous scientific review group and changes made to the project, but basing its final score on the overall merits of the current proposal.

:
No response entered

Reviewer Role: Secondary (237821)

OVERALL IMPACT
After considering all of the review criteria, summarize the significant strengths and weaknesses of the application. Will this project help establish a junior PI's research program and/or enhance an established program? Does the project have the potential for "high gain" to balance risk? Does the project scope suit the funding level and does it have the potential to exert a sustained powerful influence and/or be an important contribution to the field of SCI psychosocial research?

:
Significant strengths of the application include a clinically significant issue (physical inactivity), innovation (tailored

design), a well-designed, theory-based approach, and strong senior scientific leadership. Weaknesses of the application include a relatively junior PI, though this project will help establish a junior PI's research program. The project has the potential for "high gain" to balance risk. The project's scope is suited to the funding level and has the potential to be an important contribution to the field of SCI psychosocial research.

SIGNIFICANCE
1. Does the project address an important problem or a critical barrier in the field?

2. If the Aims of the project are achieved, how would this work change or enhance current methods, technologies, treatments, services, or interventions?

:
This project addresses an important problem or a critical barrier in the field, limited uptake of physical activity by people with SCI. In part, this stems from people with SCI being understudied with regard to effective interventions that are tailored to their needs and replicable in home environments. Data from this pilot study will inform an R01 application for funding to assess the implementation and efficacy of a full-scale SMART of this adaptive exercise intervention among people with SCI. If the Aims of the project are achieved, this work is likely to change or enhance current methods, technologies, treatments, services, and interventions

RELEVANCE
1. How is this project relevant to the mission of the Neilsen Foundation?

2. How is this project relevant to the goal of the PSR portfolio?

:
This project is relevant to the mission of the Neilsen Foundation by exploring ways to increase the physical activity of people with SCI, and in turn, improve health and quality of life of adults with SCI. This project relevant to the goal of the PSR portfolio with its behavioral focus.

INVESTIGATOR(S)
1. Are the PI, collaborators, and other contributors well suited to the project? Are relevant SCI and psychosocial research expertise represented on the proposed project team?

2. If the PI is a junior investigator, does he/she have appropriate experience, training and facilities to do the proposed work? If the PI is an established investigator, has he/she demonstrated an ongoing record of accomplishments that have advanced their field(s)?

:
The PI, collaborators, and other contributors are well suited to the project. [redacted] completed a PhD in health education [redacted] and then post-doctoral fellowships at [redacted], AL with a focus on psychosocial factors associated with adherence to exercise trials for people with physical disabilities. He is supported by a KL2 award and by [redacted] research funding. Relevant SCI and psychosocial research expertise

is represented on the proposed project team. [redacted] is director of the [redacted] and is the director of two federally funded centers, the National Center on Health, Physical Activity and Disability which has been funded by CDC since 1999, and the Rehabilitation Engineering Research Center, RecTech which has been funded by NIDILRR since 2002. The PI has appropriate experience, training and facilities to do the proposed work while his mentor and key personnel, [redacted], has demonstrated an ongoing record of accomplishments that have advanced the field.

INNOVATION
1. Does the project challenge and seek to shift current research or clinical practice/interventional paradigms by utilizing novel theoretical concepts, approaches or methodologies, instrumentation, or interventions?

2. And/or does this application apply concepts, approaches or methodologies, instrumentation, or interventions from another field of research to SCI?

3. And/or is a refinement, improvement, or new application of theoretical concepts, approaches or methodologies, instrumentation, or interventions proposed?

:
This study demonstrates innovation by pilot testing a SMART design for a telehealth exercise program for people with SCI. It applies concepts, approaches, instrumentation, or interventions from another field of research to SCI. The study seeks to increase enrollment in, adherence to, and sustainability of an exercise trial in people with SCI using an innovative study design that has not been used to evaluate exercise adherence in persons with SCI. Compared with one-size-fits-all approach to interventions developed to improve health behaviors, adaptive designs can improve adherence, and SMART designs help identify the programmatic variations that can optimize the adaptive intervention. The study uses a rigorous home-based assessments for data collection, avoiding transportation barriers.

APPROACH
1. Is the project well-suited to the Pilot stage? Are the future applicability or next steps after the Pilot work discussed (i.e., does this research help lay the groundwork for future studies or describe plans for the dissemination and translation of knowledge that will impact current practices and approaches)?

2. Are the overall strategy, methodology, and analyses well-reasoned and appropriate to accomplish the specific Aims of the project, within the proposed project period and by the project team described? Are potential problems, alternative strategies, and benchmarks for success presented?

3. If the project is in the early stages of development, will the strategy establish feasibility and will particularly risky aspects be managed adequately?

4. If the project involves clinical and/or community-based research:
a. Does the project timeline include plans for study start-up time and sufficient subject recruitment?
b. Does the start-up plan adequately address regulatory approval, if required?
c. Will the National Institute of Neurological Disorders and Stroke, Common Data Elements be used? If not, is

justification provided?

:

The project is well-suited to the Pilot stage. The applicant provides preliminary data which helps set the stage for the proposed pilot study. The design is based on social cognitive theory, and operationalizes key constructs in the study design and instrumentation. The project proceeds in a logical manner by conducting a 12-week pilot study of an intervention with 30 individuals with SCI that includes an adapted exercise program, instructor-led, individualized training, behavioral coaching, and social networking support. Participants will be randomized to either 4 weeks or 8 weeks of a novel adapted exercise program (Movement to Music [M2M]) with social networking support in order to assess the length of the first intervention stage. After the assigned period, participants with high intervention adherence will continue on the same program for the balance of the 12-weeks. Those with low adherence will be randomized to either an augmented arm that includes individualized behavioral coaching or to M2M Live, a modified version of M2M that involves one-on-one tele-exercise training, allowing for immediate feedback and accountability. The applicant will collect self-report and physiological measures to assess completion rates and conduct semi-structured interviews to assess perceived satisfaction and enjoyment. The applicant discusses the applicability of findings after the Pilot work is completed, thus laying the groundwork for future studies. The overall strategy, methodology, and analyses are well-reasoned and appropriate to accomplish the specific Aims of the project, within the proposed project period and by the project team. The applicant discusses potential problems, alternative strategies, and benchmarks for success. The proposal includes a project timeline that includes time for study start-up and sufficient subject recruitment. The start-up plan adequately addresses regulatory approval. It appears that the study will use some of the National Institute of Neurological Disorders and Stroke, Common Data Elements, though they are not identified specifically.

ENVIRONMENT
1. Will the institutional environment in which the work will be done contribute to the probability of success?

2. Are the institutional support, physical equipment and other resources available to the investigator(s) adequate for the project proposed?

3. Will the project benefit from unique features of their environment, subject populations, or collaborative arrangements?

:

The institutional environment in which the work will be done is likely to contribute to the probability of success. The institutional support, physical equipment and other resources available to the investigators appears to be adequate for the project proposed. Lakeshore Center provides a wealth of resources for the study and the investigative team has a good record of extramural funding.

NON-SCORED CRITERIA
(PLEASE NOTE THAT THE SCORE FOR THIS NON-SCORED SECTION MUST BE A "50 NON-SCORED." PLEASE DISREGARD SCORING OPTIONS 1-9 FOR THIS SECTION ONLY.)

Provide any important or relevant comments on each of the non-scored criterion below.

1. Budget

2. Ethics/Safety; protection of human subjects, including safety and data monitoring

3. Other/Additional Comments for the Applicant

4. Resubmission: When reviewing a resubmission, the committee will evaluate the application as now presented, taking into consideration the responses to comments from the previous scientific review group and changes made to the project, but basing its final score on the overall merits of the current proposal.

:
No response entered
